# Influence of Body Mass Index on Functional Capacity in Physically Active Community‐Dwelling Adult Women

**DOI:** 10.1155/jare/1948349

**Published:** 2026-01-31

**Authors:** Josivaldo de Souza-Lima, Pedro Valdivia-Moral, Gerson Ferrari, Timoteo Leandro Araujo, Sandra Mahecha-Matsudo

**Affiliations:** ^1^ Department of Didactics of Musical, Plastic and Body Expression, University of Granada, Granada, 18071, Spain, ugr.es; ^2^ Faculty of Education and Social Sciences, Universidad Andres Bello, Las Condes, Santiago, 7550000, Chile, unab.cl; ^3^ Faculty of Health Sciences, Universidad Autonoma de Chile, Santiago, Chile; ^4^ School of Physical Activity, Sports, and Health Sciences, Universidad de Santiago de Chile (USACH), Santiago, Chile, usach.cl; ^5^ Center for Studies of the Physical Fitness Research Laboratory of São Caetano do Sul, São Paulo, Brazil; ^6^ Metropolitan United Faculties, Sao Paulo, Brazil; ^7^ Center for Research in Medicine, Exercise, Sports, and Health-MEDS Clinic, Santiago, 7550000, Chile

**Keywords:** body mass index, functional capacity, handgrip strength, trunk flexibility, walking speed

## Abstract

**Background:**

Declining functional capacity is a major contributor to disability in older populations. This study aimed to examine the association between body mass index (BMI) and physical function in physically active adult women.

**Methods:**

A cross‐sectional analysis was conducted on 515 women aged 46–90 years participating in a free community‐based physical activity program in Brazil. Functional capacity was assessed using handgrip strength, trunk flexibility, lower limb muscle strength (LLMS), and walking speed. Participants were classified by BMI into underweight (< 22 kg/m^2^), eutrophic (22–27 kg/m^2^), overweight (27–30 kg/m^2^), and obese (≥ 30 kg/m^2^). One‐way ANOVA with Bonferroni post hoc tests and hierarchical multiple regression analyses were used to assess differences and associations.

**Results:**

Overweight and obese participants represented the largest proportions (27.2% and 25.6%, respectively). Walking speed was slower in obese participants (1.0 m/s) than in the eutrophic group (1.1 m/s), but this difference was not statistically significant (*p* > 0.05). Trunk flexibility was significantly lower in the obese group (21.3 cm vs. 26.3 cm, *p* < 0.05). LLMS was significantly associated with walking performance across all BMI categories.

**Conclusion:**

Higher BMI is associated with reduced flexibility and mobility in adult women. LLMS appears critical for maintaining functional independence.

## 1. Background

Population​ aging is a global phenomenon driven by increased life expectancy and decreased fertility [1, 2]. This rapid aging has led to a significant rise in the number of people who will require care in the future [3, 4]. The decline in functional capacity is a common cause of the caregiving burden among the aging population [5]. Improving functional capacity is a crucial step in shifting from a disease‐centered paradigm to a functionality‐centered one, facilitating healthy aging [6–8]. While body mass index (BMI) and functional capacity have been widely studied, few studies have focused on physically active older women, limiting the understanding of how BMI affects functionality in this subgroup.

Functional capacity in adult women is influenced by multiple factors, including BMI. Studies have shown that a higher BMI is negatively associated with mobility and walking speed in adult women. For instance, a study by Houston et al. [9] found that an elevated BMI is associated with poorer functional capacity and a higher risk of disability in this population [9]. Additionally, the health, aging, and body composition study showed that both overweight and obese men and women aged 70–79 have a higher risk of mobility limitation. The hazard ratios for obesity were 1.61 (95% CI: 1.28–2.02) in men and 2.14 (95% CI: 1.75–2.62) in women [10].

BMI has been linked to various health and functional measures in this population, impacting independence and quality of life. An elevated BMI is associated with poorer health‐related quality of life (HRQoL), both physically and mentally, with significant decreases in HRQoL scores. Obesity has been associated with reduced HRQoL in older adults [11]. Another study conducted in Brazil found that 10.5% of obese individuals (BMI 30–34.9 kg/m^2^) and 11.0% of those with morbid obesity (BMI ≥ 35 kg/m^2^) had sarcopenia, compared to only 3.8% of individuals with a eutrophic BMI (18.5–24.9 kg/m^2^). Sarcopenia is a crucial indicator of functionality [12].

Muscular strength, particularly lower limb muscle strength (LLMS), is a critical determinant of mobility in adult women. Improving overall body strength can benefit walking speed in certain BMI groups [13–15].

A study by Latham et al. [16] conducted a randomized controlled trial with a sample of frail adult women, demonstrating that a progressive resistance training program improved muscle strength and mobility. Participants with an initial BMI of 30 kg/m^2^ or more experienced a 30% increase in LLMS and an improvement in the walking speed of 0.12 m/s, compared to those who did not receive the intervention [16].

Differences in functional capacity according to BMI can provide valuable insights for designing personalized intervention programs. These programs can focus on improving specific areas of functional capacity, which could reduce the caregiving burden and promote healthy aging. The participants are adult women involved in the community with a free physical activity program. This study aims to explore how BMI influences various physical capacities, including grip strength, flexibility, LLMS, and balance, in adult women. Additionally, it suggests specific approaches to improve health and functionality by addressing the identified impacts of BMI on these key physical capacities.

Recent evidence also connects functional fitness, including grip strength and balance, with work ability in older adults, supporting the relevance of preserving these capacities beyond BMI‐related risks [17].

This study stands out by focusing on physically active older women, a population often underrepresented in gerontological research. Unlike sedentary or frail populations, physically active individuals may present different patterns of functional decline, which has important implications for preventive public health strategies.

## 2. Methods

### 2.1. Study Design and Sample

The study included a sample of 515 women aged 46–90 years (mean age: 67.4 ± 0.3 years) who participated in physical activities offered by a Community Center in Brazil. The initial sample size was larger; however, only these 515 women had complete data for all variables of interest required for the analyses. These activities were conducted three times a week, with a 50‐min session, which included aerobic exercises, stretching, flexibility, and balance, all guided by a physical education professional. The sample was selected using nonprobability convenience sampling from a Research Longitudinal Project on Aging and Physical Fitness conducted since 1997. Participants were enrolled sequentially as they arrived at the community program sessions. Eligibility screening included verification of age, community‐dwelling status, and the ability to ambulate independently. Only participants with complete assessments were included in the final analytic sample. Full details of the Research Longitudinal Project on Aging and Physical Fitness design are reported elsewhere [18, 19].

Inclusion criteria were female, age over 46 years of age, no medical restrictions for physical activities, and participation in at least 75% of the classes. Informed consent was obtained from each participant before the start of the study for conducting the evaluations and using the obtained data. The Research Longitudinal Project on Aging and Physical Fitness was approved by the Ethics Committee of the Municipal Health Foundation of the Municipality of São Caetano do Sul (number: 028/2010‐A). Participants gave informed consent to participate in the study before taking part.

### 2.2. BMI

BMI was calculated as weight (kg) divided by height squared (m^2^), was calculated using a calibrated digital scale and stadiometer, ensuring precision in anthropometric assessments. Weight was measured using a digital scale, and height was measured with a stadiometer. The participants were subdivided into groups according to their BMI: underweight: < 22 kg/m^2^, eutrophic: 22 < 27 kg/m^2^, overweight: 27 < 30 kg/m^2^, and obese: ≥ 30 kg/m^2^, using classifications adapted from Gallagher et al. [20], who proposed alternative BMI thresholds better suited for older adults to reflect age‐related changes in body composition [20]. These thresholds have been applied in geriatric populations to improve sensitivity in identifying functional impairments.

### 2.3. Functional Capacity

Handgrip strength was assessed using a TKK 5001 hand dynamometer, following a widely adopted standardized protocol. Participants were evaluated in a seated position with the shoulder in neutral rotation, elbow flexed at 90°, forearm in a neutral position, and wrist between 0° and 30° of extension, and 0°–15° of ulnar deviation. Two warm‐up trials were allowed, followed by three maximal attempts with the dominant hand, alternating with 30–60 s of rest to minimize fatigue. Verbal encouragement was provided to ensure maximal voluntary contraction. The best value of the three trials was recorded. This protocol has been shown to improve measurement reproducibility in aging populations [21].

### 2.4. Flexibility Assessment

Trunk flexibility was measured using the sit‐and‐reach test, a widely recognized and validated method in the literature for assessing flexibility in older adults. Participants sat on the floor with their legs extended and performed a forward bend, attempting to reach the farthest distance possible with their hands without bending their knees. The distance reached was measured in centimeters, and the best value from three consecutive attempts was recorded. This procedure was conducted following the standard protocols described in previous studies [22].

LLMS was assessed using the chair stand test. Participants were asked to sit on a chair approximately 45 cm high with their arms crossed over their chest and perform as many repetitions as possible within 30 s. The test was conducted three times, and the highest number was recorded. This protocol is based on previous studies that have validated its use for measuring LLMS in adult women [19, 23]

### 2.5. Balance

The one‐leg stance (OLS) test was performed following established clinical protocols. Participants stood barefoot on their preferred leg, with hands on their hips and the nonsupporting knee flexed at approximately 90°. The timer started when the foot left the floor and stopped when the foot touched the ground again or when 30 s was reached. Each participant completed three attempts, and the best performance was used for analysis. The 30 s ceiling and three‐attempt structure are widely used in functional aging research and help minimize measurement variability. To reduce fatigue effects, a 30–60 s rest was provided between attempts.

### 2.6. Walking and Speed Performance

Walking performance was evaluated using a gait speed test. Participants walked a predetermined distance of 3.33 m at both their usual and maximum speed. The speed was measured in meters per second (m/s). Participants performed the test three times, and the best result was recorded. This protocol follows the guidelines from previous research on gait speed assessment in older adults [19].

### 2.7. Statistical Analysis

Data are presented as means and 95% confidence intervals (CIs). A one‐way analysis of variance (ANOVA) with Bonferroni post hoc test was used to compare subjects grouped by BMI categories (underweight: < 22 kg/m^2^, eutrophic: 22 < 27 kg/m^2^, overweight: 27 < 30 kg/m^2^, and obese: ≥ 30 kg/m^2^). Associations between functional capacity and walking performance at the usual and maximum speed were investigated using univariate and hierarchical multiple regression analyses. In the hierarchical regression analysis, models were adjusted to account for potential covariates, and *β* values and 95% CIs were calculated. Pearson correlation analysis was used to assess the relationship between walking performance (usual and maximum speed) and functional capacities (grip strength, trunk flexibility, LLMS, and balance) across different BMI categories. Significant correlations are indicated with *p* < 0.05 and *p* < 0.01.

Assumptions of normality and homoscedasticity were evaluated using the Shapiro–Wilk and Levene’s test, respectively, to ensure the validity of ANOVA and regression analyses.

Scatter plots were generated to illustrate the relationship between functional capacities (grip strength, trunk flexibility, LLMS, and balance) and walking performance (speed and usual walking speed) for the total sample. Pearson correlation coefficients (*r*) were calculated to assess the strength and direction of these associations, with significant correlations indicated at *p* < 0.05 and *p* < 0.01. *p* values < 0.05 were considered statistically significant for all tests (two‐tailed). Additionally, potential interactions between independent variables were explored, and sensitivity analyses were conducted to evaluate the robustness of the results. All analyses were performed using Predictive Analytics Software (PASW) 17.0 for Windows (PASW, Inc., Chicago, IL).

This study was reported in accordance with the STROBE (Strengthening the Reporting of Observational Studies in Epidemiology) guidelines. A completed STROBE checklist is provided as Supporting Information.

## 3. Results

In the study sample of 515 women, the majority were classified as eutrophic (38.83%, BMI 22–27 kg/m^2^) and overweight (27.18%, BMI 27–30 kg/m^2^) (Table 1).

**TABLE 1:** General (mean and 95%CI) characteristics and functional capacity according to body mass index.

	**Body mass index (kg·m** ^**2**^ **)**				
	**Underweight (< 22 kg/m** ^**2**^ **; *N* = 43)** ^**a**^	**Eutrophic (22–27 kg/m** ^**2**^ **; *N* = 200)** ^**b**^	**Overweight (> 27–30 kg/m** ^**2**^ **; *N* = 140)** ^**c**^	**Obese (> 30 kg/m** ^**2**^ **; *N* = 132)** ^**d**^	**Total sample (*N* = 515)**

Age (yrs.)	66.9 (53.0–82.0)	68.3 (47.0–90.0)	67.3 (46.0–85.0)	66.3 (47.0–84.0)	67.4 (46.0–90.0)
Body mass (kg)	48.6 (33.8–60.5)^bcd^	61.5 (47.3–80.0)^cd^	69.7 (54.9–87.6)^d^	80.4 (60.9–114.2)	67.6 (33.8–114.2)
Body height (m)	1.55 (1.42–1.72)	1.57 (1.41–1.84)^d^	1.56 (1.41–1.75)^d^	1.54 ± 0.06 (1.05–1.70)	156.1 (105.1–180.4)
Body mass index (kg/m^2^)	20.1 (14.6–21.9)^bcd^	24.9 (22.1–27.0)^cd^	28.4 (26.8–30.0)^d^	33.7 (30.1–55.2)	27.8 (14.6–55.2)
Walking performance					
Walking (s)	3.0 (2.1–6.5)	3.2 (1.7–5.7)	3.2 (2.0–9.7)	3.4 (1.2–5.9)	3.2 (1.2–9.7)
Speed (m·s^−1^)	1.1 (0.5–1.6)	1.1 (0.6–1.9)	1.1 (0.3–1.6)	1.0 (0.6–2.9)	1.1 (0.3–2.9)
Functional capacity					
Grip strength (kgf)	23.9 (16.0–41.0)	26.4 (12.0–52.0)	25.8 (14.0–45.0)	25.4 (12.0–50.0)	25.8 (12.0–52.0)
Flexibility (cm)	26.2 (9.0–75.0)	26.3 (3.0–89.0)^d^	23.4 (6.0–70.0)	21.3 (1.0–84.0)	24.2 (1.0–89.0)
Lower limb muscle strength (rep)	14.5 (6.0–24.0)	15.3 (3.0–33.0)^d^	15.1 (5.0–40.0)^d^	12.8 (4.0–26.0)	14.6 (3.0–40.0)
Balance (s)	16.6 (0.2–30.0)	18.3 (0.3–30.0)^d^	15.5 (0.2–30.0)	12.0 (0.0–48.1)	15.8 (0.0–48.1)

*Note:* Data are presented as means with 95% confidence intervals. Comparisons between BMI groups were conducted using one‐way ANOVA with Bonferroni post hoc test. General characteristics and functional capacity measures across body mass index (BMI) categories in adult women participating in a community‐based physical activity program. Values are expressed as means and 95% confidence intervals. Comparisons between BMI groups were performed using one‐way ANOVA with Bonferroni post hoc tests. Superscript letters indicate statistically significant differences (*p* < 0.05) between groups.

In the analysis of functional capacity, handgrip strength varied significantly between BMI groups. The average strength was highest in the eutrophic group (26.4 kgf) and lowest in the underweight group (23.9 kgf). The overweight and obese groups showed averages of 25.8 and 25.4 kgf, respectively (Table 1). Trunk flexibility and LLMS also showed significant differences between the groups, with lower flexibility and balance observed in the groups with higher BMI, while LLMS tended to be higher.

The correlations between walking performance (walking and speed) and functional capacities are presented in Table 2. Handgrip strength showed weak correlations with walking performance across all BMI groups, with few significant correlations. Flexibility showed significant correlations with walking speed in the BMI group of eutrophics. LLMS exhibited strong significant correlations with walking performance in most BMI groups, indicating its importance for mobility. Balance also demonstrated significant correlations with walking performance in several groups, especially those with higher BMI.

**TABLE 2:** Correlation between walking performance and functional capacity according to body mass index.

	**Body mass index**									
	**Underweight (< 22 kg/m** ^**2**^ **)**		**Eutrophic (22 < 27 kg/m** ^**2**^ **)**		**Overweight (27 < 30 kg/m** ^**2**^ **)**		**Obese (≥ 30 kg/m** ^**2**^ **)**		**Total sample**	
	**Walking**	**Speed**	**Walking**	**Speed**	**Walking**	**Speed**	**Walking**	**Speed**	**Walking**	**Speed**

Grip strength	−0.14	0.11	−0.10	0.12	−0.01	0.03	−0.11	0.08	−0.08	0.09^*p*<0.05^
Flexibility	−0.17	0.32^*p*<0.05^	−0.23^*p*<0.01^	0.21^*p*<0.01^	0.03	−0.02	−0.08	0.10	−0.13^*p*<0.01^	0.15^*p*<0.01^
LLMS	−0.53^*p*<0.01^	0.50^*p*<0.01^	−0.29^*p*<0.01^	0.31^*p*<0.01^	−0.28^*p*<0.01^	0.33^*p*<0.01^	−0.33^*p*<0.01^	0.25^*p*<0.05^	−0.31^*p*<0.01^	0.31^*p*<0.01^
Balance	−0.03	−0.04	−0.30^*p*<0.01^	0.28^*p*<0.01^	−0.22^*p*<0.05^	0.21^*p*<0.05^	−0.29^*p*<0.01^	0.19	−0.26^*p*<0.01^	0.22^*p*<0.01^

*Note:* Correlation coefficients between walking performance (walking and speed) and functional capacity variables (grip strength, flexibility, lower limb muscle strength [LLMS], and balance), stratified by body mass index (BMI) categories.

*p* < 0.05; *p* < 0.01.

The multiple regression analyses (Table 3) indicated that age and body mass have a significant impact on various measures of functional capacity, and this impact varies according to BMI (walking performance (walking and speed)).

**TABLE 3:** Beta coefficients (*p* values) from multiple regression analyses examining the association between functional capacity and walking performance (walking speed) across different BMI categories.

Body mass index	Walking				Speed			
	Grip strength	Flexibility	LLMS	Balance	Grip strength	Flexibility	LLMS	Balance
Underweight (< 22 kg/m^2^)								
Age	−0.058 (0.620)	−0.135 (0.434)	−0.037 (0.819)	−0.348 (0.077)	−0.051 (0.661)	−0.140 (0.393)	−0.082 (0.605)	−0.348 (0.074)
Body mass	1.110 (0.0005)	−0.017 (0.947)	0.508 (0.038)	0.091 (0.745)	1.095 (0.0005)	−0.016 (0.946)	0.584 (0.015)	0.091 (0.737)
Body mass index	−0.791 (0.0005)	0.178 (0.466)	−0.507 (0.034)	−0.023 (0.932)	−0.778 (0.0005)	0.184 (0.426)	−0.558 (0.018)	−0.025 (0.926)
Walking or Speed	0.032 (0.788)	−0.165 (0.338)	−0.442 (0.010)	0.034 (0.859)	0.021 (0.852)	0.318 (0.052)	0.447 (0.007)	−0.069 (0.711)
Eutrophic (22 < 27 kg/m^2^)								
Age	0.068 (0.249)	0.070 (0.314)	0.053 (0.480)	0.013 (0.867)	0.064 (0.280)	0.063 (0.363)	0.042 (0.579)	0.004 (0.957)
Body mass	0.646 (0.0005)	−0.120 (0.139)	0.346 (0.000)	0.007 (0.940)	0.644 (0.0005)	−0.124 (0.128)	0.340 (0.0005)	0.002 (0.986)
Body mass index	−0.346 (0.0005)	−0.024 (0.766)	−0.306 (0.001)	−0.051 (0.574)	−0.345 (0.0005)	−0.022 (0.790)	−0.303 (0.001)	−0.048 (0.601)
Walking or Speed	−0.075 (0.205)	−0.227 (0.001)	−0.282 (0.0005)	−0.299 (0.000)	0.089 (0.133)	0.212 (0.003)	0.295 (0.0005)	0.284 (0.0005)
Overweight (27 < 30 kg/m^2^)								
Age	−0.042 (0.573)	0.001 (0.994)	−0.191 (0.059)	−0.085 (0.394)	−0.037 (0.618)	0.002 (0.979)	−0.196 (0.048)	−0.095 (0.341)
Body mass	0.565 (0.0005)	−0.018 (0.849)	0.091 (0.396)	0.099 (0.354)	0.562 (0.0005)	−0.019 (0.841)	0.084 (0.421)	0.099 (0.354)
Body mass index	−0.181 (0.025)	0.092 (0.331)	−0.078 (0.475)	−0.074 (0.492)	−0.175 (0.031)	0.094 (0.321)	−0.061 (0.571)	−0.073 (0.499)
Walking or Speed	0.043 (0.571)	0.014 (0.872)	−0.232 (0.025)	−0.192 (0.061)	−0.010 (0.890)	−0.003 (0.969)	0.298 (0.004)	0.185 (0.069)
Obese (≥ 30 kg/m^2^)								
Age	0.009 (0.903)	0.076 (0.393)	0.123 (0.219)	0.182 (0.072)	0.010 (0.902)	0.076 (0.393)	0.125 (0.224)	0.183 (0.077)
Body mass	0.559 (0.0005)	−0.031 (0.779)	0.205 (0.097)	0.069 (0.575)	0.564 (0.0005)	−0.027 (0.805)	0.235 (0.062)	0.099 (0.428)
Body mass index	−0.063 (0.506)	−0.222 (0.042)	0.025 (0.834)	0.127 (0.299)	−0.066 (0.480)	−0.223 (0.040)	0.001 (0.994)	0.101 (0.419)
Walking or Speed	−0.073 (0.348)	−0.066 (0.457)	−0.317 (0.002)	−0.293 (0.004)	0.070 (0.366)	0.076 (0.390)	0.241 (0.019)	0.198 (0.055)
Total sample								
Age	0.032 (0.409)	0.059 (0.172)	0.015 (0.761)	0.027 (0.578)	0.030 (0.440)	0.054 (0.209)	0.003 (0.954)	0.017 (0.726)
Body mass	0.910 (0.0005)	−0.073 (0.368)	0.335 (0.0005)	0.054 (0.561)	0.909 (0.0005)	−0.072 (0.375)	0.342 (0.0005)	0.065 (0.485)
Body mass index	−0.721 (0.0005)	−0.123 (0.133)	−0.400 (0.0005)	−0.177 (0.057)	−0.717 (0.0005)	−0.123 (0.132)	−0.407 (0.0005)	−0.193 (0.040)
Walking or Speed	−0.046 (0.235)	−0.115 (0.009)	−0.284 (0.0005)	−0.243 (0.000)	0.065 (0.096)	0.127 (0.004)	0.285 (0.0005)	0.202 (0.0005)

*Note:* Beta coefficients (*β*) and significance levels (*p* values) from hierarchical multiple regression analyses examining the association between walking performance (walking speed) and functional capacity variables (grip strength, flexibility, lower limb muscle strength [LLMS], and balance) across BMI categories.

Figure 1 provides a visual representation of these relationships, showing that walking speed and performance are significantly correlated with grip strength, flexibility, LLMS, and balance; walking speed is associated with greater grip strength and LLMS, as well as improved flexibility and balance.

FIGURE 1Scatter plot for functional capacity according to walking performance (speed [a–d]; walking [e–g]) for total sample. (a) Grip strength by speed. (b) Flexibility by speed. (c) LLMS by speed. (d) Balance by speed. (e) Flexibility by walking. (f) LLMS by walking. (g) Balance by walking.

**Figure:**
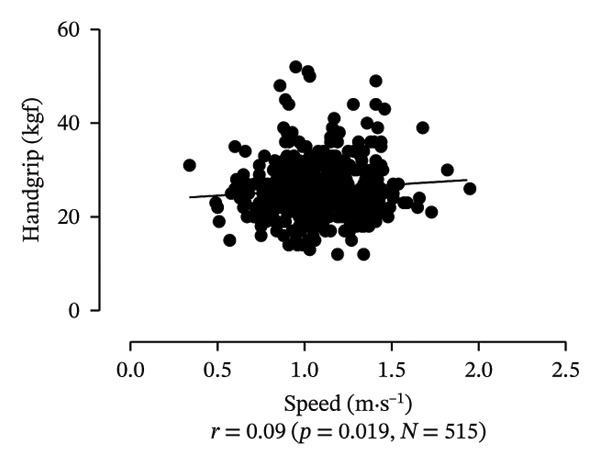
(a)

**Figure:**
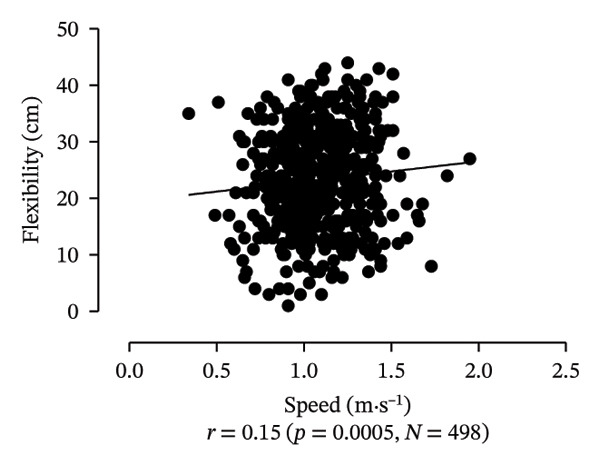
(b)

**Figure:**
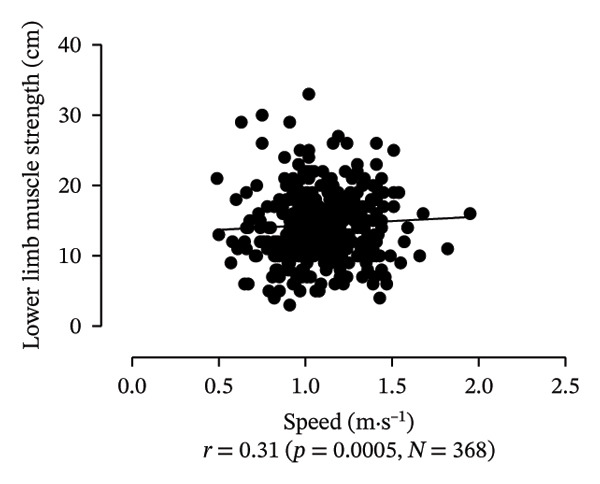
(c)

**Figure:**
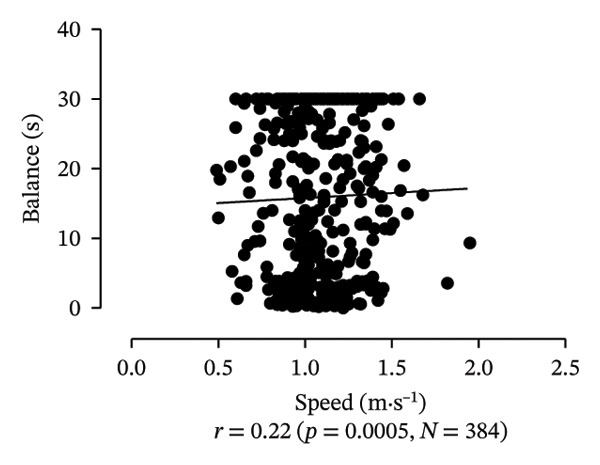
(d)

**Figure:**
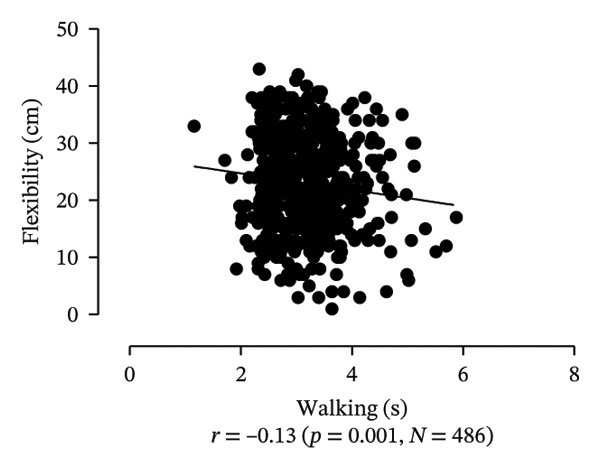
(e)

**Figure:**
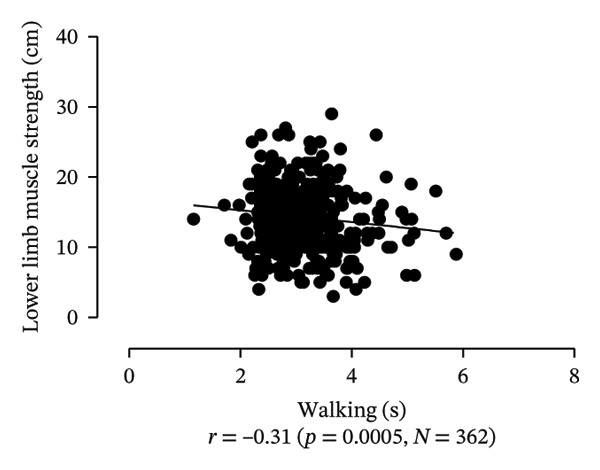
(f)

**Figure:**
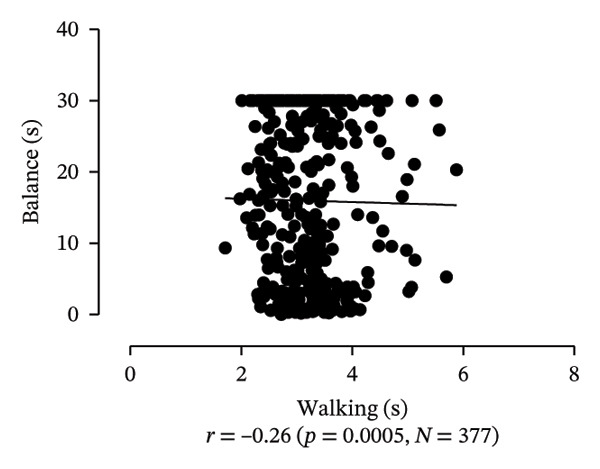
(g)

## 4. Discussion

The primary objective of this study was to examine the association between BMI and functional capacity in adult women engaged in a regular physical activity program. We aimed to determine how different levels of BMI influence key physical capacities, including walking speed, grip strength, flexibility, and LLMS. By exploring these relationships, our goal was to identify specific approaches that could be employed to enhance health and functionality in this population, thereby providing valuable insights for designing targeted interventions that promote healthy aging.

In our study, the average walking speed was 1.0 m/s for those with a BMI > 30 kg/m^2^, compared to 1.1 m/s in the eutrophic group; however, this difference was not statistically significant (*p* > 0.05). The reduction in walking speed among those with a higher BMI is concerning, as walking speed is a crucial indicator of functional capacity and is associated with greater independence and a better quality of life in old age. Previous studies have shown that a decrease in walking speed is strongly correlated with an increased risk of falls, hospitalization, and mortality [24–27]. Although the reduction in walking speed was not statistically significant, a decrease of 0.1 m/s is considered clinically meaningful and has been associated with greater risks of mobility limitation and dependence in older adults.

These age‐specific BMI categories provide a more accurate classification of body composition changes associated with aging, allowing for a better understanding of functional outcomes compared to using the standard WHO thresholds.

In the study by Stenholm et al. [28], it was observed that obese older adults with low muscle strength had a significantly lower initial walking speed of approximately 0.9 m/s and experienced a more pronounced decline in walking speed over a 6 year follow‐up period. Additionally, this group showed a higher risk of developing new mobility disabilities, particularly among those under 80 years old. The combination of obesity and low muscle strength not only impacted walking speed but also significantly increased the risk of mobility disability and other adverse health events [28].

Elevated BMI also affected handgrip strength. Obese participants had an average handgrip strength of 25.4 kgf, compared to 26.4 kgf in the eutrophic group; however, this difference was not statistically significant (*p* > 0.05). Handgrip strength is an important predictor of overall health and functional capacity in older adults, and its decline may reflect a loss of muscle mass and general strength. A study conducted by de Souza Moreira et al. [29] in the Longitudinal Study of Aging in Brazil (ELSI‐Brazil) supports these findings, showing that overweight and obesity are inversely associated with muscle weakness. In this study, overweight men had a lower probability of muscle weakness (odds ratio [OR] 0.66, 95% CI 0.52–0.83) and those with obesity had an even lower probability (OR 0.49, 95% CI 0.31–0.78).

Similarly, obese women showed a lower probability of muscle weakness (OR 0.69, 95% CI 0.52–0.92). These results suggest that while a high BMI may be associated with a reduction in walking speed, it does not necessarily have the same negative effect on handgrip strength. This finding implies a potential protective effect of elevated BMI on hand muscle strength, possibly due to greater muscle mass in individuals with higher BMI, although this does not directly translate into better overall functional capacity due to other obesity‐related factors [29]. Regression analysis indicated that age and BMI significantly predicted LLMS and flexibility outcomes (e.g., *β* = −0.21, 95% CI: −0.32 to −0.11), confirming their independent roles in functional capacity.

In terms of flexibility, participants with elevated BMI showed a flexibility of 21.3 cm, significantly lower than the 26.3 cm observed in the eutrophic group. Flexibility is crucial for performing daily activities and preventing falls. Additionally, balance was also affected, with a notable reduction in balance time for participants with elevated BMI. These decreases in flexibility and balance can increase the risk of falls and injuries, further limiting the independence of older adults.

These findings are consistent with the study conducted by Vagetti et al. [30], which found that older obese women had significantly lower performance in flexibility tests compared to eutrophic women. In this study, trunk flexibility was measured using the sit‐and‐reach test, and results showed that obese women had an average flexibility of 19.3 cm, compared to 23.9 cm in the eutrophic group. The OR for low flexibility in the obese group was 1.96 (95% CI: 1.52–2.53), highlighting the strong association between elevated BMI and decreased flexibility.

Additionally, Vagetti et al. [30] study also demonstrated that balance was also affected in women with elevated BMI. The balance test revealed that obese women had significantly lower balance time compared to their eutrophic counterparts, which aligns with our findings. This underscores the importance of maintaining a healthy BMI to preserve both flexibility and balance in old age [30].

Despite our sample consisting of adult women who were already engaged in physical activities, BMI emerged as a critical factor affecting several aspects of physical and functional capacity. For instance, participants with a BMI greater than 30 kg/m^2^ had an average walking speed of 1.0 m/s, compared to 1.1 m/s in the eutrophic group. This reduction in walking speed is concerning, as it is strongly correlated with a higher risk of falls, hospitalization, and mortality [28]. Additionally, handgrip strength was also affected, averaging 25.4 kgf in the elevated BMI group compared to 26.4 kgf in the eutrophic group. Flexibility, measured in centimeters, was 21.3 cm in the elevated BMI group, significantly lower than the 26.3 cm observed in the eutrophic group [30].

According to Oppert et al. [31], physical training programs that combine aerobic and resistance exercises are recommended to improve body composition, cardiometabolic fitness, and muscle strength in overweight and obese adults. These programs should not only focus on weight loss but also on enhancing physical fitness and quality of life. Therefore, it is crucial that physical activity programs for adult women consider the intensity and type of exercise, as well as to customize the program based on BMI and individual capacities: These findings underscore the need for public health initiatives that include BMI‐informed exercise prescriptions to mitigate functional decline in overweight and obese women. Another limitation is the lack of data on comorbidities and medication use, which could influence both BMI and functional outcomes. Future studies should control these variables to better isolate the effects of BMI on functional capacity.

Programs that integrate aerobic and resistance training tailored to BMI could enhance mobility, prevent disability, and promote independence in this growing population segment. This may include supervised high‐intensity training and strategies to reduce sedentary behavior to maximize health benefits [31].

This study presents both limitations and strengths. One key limitation is the reliance on BMI as the primary measure of body composition, rather than more precise assessments such as skinfold thickness and circumferences, which were available in our dataset. Additionally, the cross‐sectional design limits our ability to establish definitive causal relationships between BMI and functional capacity variables. Another limitation is that the sample consisted exclusively of adult women engaged in regular physical activity; however, their specific level of physical activity was not considered, which may have influenced the results. Furthermore, the findings cannot be extrapolated to older men, as sex‐related physiological differences may play a role in functional capacity outcomes. This study also carries a potential selection bias, as individuals who participate in structured physical activity programs are likely to be healthier and more active than the general population. Additionally, we did not control other potentially influential factors, such as comorbidities, medication use, and socioeconomic status, which could impact both BMI and functional capacity. Lastly, although functional capacity was assessed using standardized physical tests, these measures may not fully capture all dimensions of functionality in adults. Future research should adopt a longitudinal approach, incorporate objective assessments of physical activity levels, and include both sexes to provide a more comprehensive understanding of the impact of BMI on functional capacity.

Emerging strategies like mobile health (mHealth) interventions have also shown promise in promoting lifestyle changes and functional capacity in older adults, complementing physical activity programs [32].

Our focus on physically active older women offers valuable insights into a population that differs significantly from the more commonly studied sedentary or frail groups, highlighting the importance of tailored preventive programs based on activity level.

## 5. Conclusions

We conclude that BMI is significantly associated with key indicators of functional capacity, namely, walking speed, handgrip strength, and flexibility in physically active adult women. Those with a BMI ≥ 30 kg/m^2^ demonstrated lower performance in these domains, which may affect their mobility and autonomy. Among these, LLMS showed the strongest correlation with walking speed, emphasizing its critical role in maintaining physical functionality in this population.

**Nomenclature**
BMI: Body Mass Index
LLMS: Lower Limb Muscle Strength
kgf: Kilogram‐force
PA: Physical Activity
CI: Confidence Interval
OR: Odds Ratio

## Author Contributions

Josivaldo de Souza‐Lima designed the study, coordinated data collection, and drafted the manuscript. Gerson Ferrari, Pedro Valdivia‐Moral, and Timoteo Leandro Araujo assisted with statistical analysis and interpretation. Sandra Mahecha‐Matsudo provided critical revisions and conceptual guidance.

## Funding

This research did not receive any specific grant from funding agencies in the public, commercial, or not‐for‐profit sectors. Funding for open access publishing was provided by Universidad de Granada/CBUA; Scientific publication support was enabled and organized by the University of Granada Library.

## Disclosure

All authors reviewed and approved the final manuscript.

## Ethics Statement

The Research Ethics Committee (approval no. 028/2010‐A) approved the study. All participants provided written informed consent.

## Consent

The authors have nothing to report.

## Conflicts of Interest

The authors declare no conflicts of interest.

## Supporting Information

STROBE Checklist.

## Supporting information


**Supporting Information** Additional supporting information can be found online in the Supporting Information section.

## Data Availability

The data that support the findings of this study are available on request from the corresponding author. The data are not publicly available due to privacy or ethical restrictions.
