# Supplementary material for: Influence of Body Mass Index on Functional Capacity in Physically Active Community‐Dwelling Adult Women
Source: J Aging Res. 2026 Jan 31;2026:1948349. doi: 10.1155/jare/1948349 (PMC12859525; doi:10.1155/jare/1948349)
Supplement: Supplementary file 1 — Supporting Information Additional supporting information can be found online in the Supporting Information section. [file JARE-2026-1948349-s001.docx]

**STROBE CHECKLIST — Cross-sectional Study**

**Study:** *Sedentary Behavior and Hip Joint Mobility in University Students: A Sex-Based Analysis*
**Design:** Cross-sectional observational study

**FULL STROBE**

| **STROBE Item** | **Recommendation** | **Where it appears in the manuscript** |
| --- | --- | --- |
| **1. Title and abstract** | Indicate the study design; provide an informative and balanced abstract. | Title page; Abstract. |
| **2. Background/rationale** | Explain the scientific background and rationale. | Introduction, paragraphs 1–4. |
| **3. Objectives** | State specific objectives and prespecified hypotheses. | Last paragraph of Introduction. |
| **4. Study design** | Present key elements of study design at the beginning. | Methods – “Study design and setting”. |
| **5. Setting** | Describe setting, location, and relevant dates, including periods of data collection. | Methods – “Study design and setting”; “Data collection”. |
| **6. Participants** | Give eligibility criteria and methods of selection. | Methods – “Participants”. |
| **7. Variables** | Clearly define outcomes, exposures, predictors, confounders. | Methods – ROM measurements, Sedentary time assessment. |
| **8. Data sources/measurement** | Provide measurement details for each variable. | Methods – ROM protocol; goniometer reliability; IPAQ sitting-time items. |
| **9. Bias** | Describe efforts to address potential biases. | Methods – standardization, single trained examiner; Discussion – limitations. |
| **10. Study size** | Explain how study size was determined. | Methods – “Sample size estimation”. |
| **11. Quantitative variables** | Explain handling of quantitative variables. | Methods – “Statistical analysis”. |
| **12. Statistical methods** | Describe all statistical methods, including handling of confounding, subgroup analyses, and sensitivity analyses. | Methods – “Statistical analysis”. |
| **13. Participants** | Report numbers at each stage, including eligible, included, and analyzed. | Results – opening paragraph; Table 1. |
| **14. Descriptive data** | Provide characteristics of study participants and information on missing data. | Results – Table 1 (complete dataset; no missing data). |
| **15. Outcome data** | Report numbers of outcome events or summary measures. | Results – Table 2; Figure 1. |
| **16. Main results** | Provide unadjusted estimates, confidence intervals, and specify which confounders were adjusted for. | Results – Table 2 (mean differences + 95% CI). |
| **17. Other analyses** | Report subgroup analyses and additional analyses. | Results – sex-based analyses; correlation analysis. |
| **18. Key results** | Summarize key results with reference to objectives. | Discussion – first paragraph. |
| **19. Limitations** | Discuss limitations, potential biases, and their direction and magnitude. | Discussion – “Limitations” section. |
| **20. Interpretation** | Provide cautious overall interpretation considering other evidence. | Discussion – integrative paragraphs. |
| **21. Generalizability** | Discuss external validity or generalizability. | Discussion – final paragraphs. |
| **22. Funding** | Give the source of funding and the role of funders. | Funding statement. |
